# The Role of Lamins in the Nucleoplasmic Reticulum, a Pleiomorphic Organelle That Enhances Nucleo-Cytoplasmic Interplay

**DOI:** 10.3389/fcell.2022.914286

**Published:** 2022-06-16

**Authors:** Merel Stiekema, Frederik Houben, Fons Verheyen, Marcel Borgers, Julia Menzel, Martin Meschkat, Marc A. M. J. van Zandvoort, Frans C. S. Ramaekers, Jos L. V. Broers

**Affiliations:** ^1^ Department of Genetics and Cell Biology, Maastricht University Medical Centre, Maastricht, Netherlands; ^2^ GROW-School for Oncology and Reproduction, Maastricht University Medical Centre, Maastricht, Netherlands; ^3^ Department of Healthcare, PXL University College, Hasselt, Belgium; ^4^ Abberior Instruments GmbH, Göttingen, Germany; ^5^ CARIM-School for Cardiovascular Diseases, Maastricht University Medical Centre, Maastricht, Netherlands; ^6^ Institute for Molecular Cardiovascular Research IMCAR, RWTH Aachen University, Aachen, Germany

**Keywords:** nuclear invaginations, nucleoplasmic reticulum, lamins, calcium regulation, electron microscopy, STED microscopy

## Abstract

Invaginations of the nuclear membrane occur in different shapes, sizes, and compositions. Part of these pleiomorphic invaginations make up the nucleoplasmic reticulum (NR), while others are merely nuclear folds. We define the NR as tubular invaginations consisting of either both the inner and outer nuclear membrane, or only the inner nuclear membrane. Specifically, invaginations of both the inner and outer nuclear membrane are also called type II NR, while those of only the inner nuclear membrane are defined as type I NR. The formation and structure of the NR is determined by proteins associated to the nuclear membrane, which induce a high membrane curvature leading to tubular invaginations. Here we review and discuss the current knowledge of nuclear invaginations and the NR in particular. An increase in tubular invaginations of the nuclear envelope is associated with several pathologies, such as laminopathies, cancer, (reversible) heart failure, and Alzheimer’s disease. Furthermore, viruses can induce both type I and II NR. In laminopathies, the amount of A-type lamins throughout the nucleus is generally decreased or the organization of lamins or lamin-associated proteins is disturbed. Also, lamin overexpression or modulation of lamin farnesylation status impacts NR formation, confirming the importance of lamin processing in NR formation. Virus infections reorganize the nuclear lamina *via* (de)phosphorylation of lamins, leading to an uneven thickness of the nuclear lamina and in turn lobulation of the nuclear membrane and the formation of invaginations of the inner nuclear membrane. Since most studies on the NR have been performed with cell cultures, we present additional proof for the existence of these structures *in vivo*, focusing on a variety of differentiated cardiovascular and hematopoietic cells. Furthermore, we substantiate the knowledge of the lamin composition of the NR by super-resolution images of the lamin A/C and B1 organization. Finally, we further highlight the essential role of lamins in NR formation by demonstrating that (over)expression of lamins can induce aberrant NR structures.

## 1 Introduction

The term nucleoplasmic reticulum (NR), first launched by Echevarría et al. (2003) was defined as a nuclear invagination of both the inner nuclear membrane (INM) and outer nuclear membrane (ONM), including the underlying nuclear lamina (Fricker et al., 1997; Clubb and Locke, 1998; Broers et al., 1999; Echevarría et al., 2003). These invaginations form branched tubular structures throughout the whole nucleus that contain many of the different elements normally only present in the cytoplasm, such as filamentous cytoskeletal structures. The presence of these invaginations has long been regarded as a non-physiological structure or a technical artefact due to cell culturing or chemical fixation. Indeed, the presence of this intranuclear structure challenged the classical view of the nucleus as a structure with a smooth and continuous INM. However, different types of fixation and live cell imaging confirmed the existence of the NR, excluding the possibility that the NR can be generated as a consequence of fixation (Bridger et al., 1993; Broers et al., 1999). Studies performed since the first description of the NR have led to increased evidence for the existence of the NR as a genuine intranuclear structure.

In this study we review and discuss the current knowledge of nuclear invaginations and the NR in particular. So far, most studies on the NR have been performed with cell cultures. We present additional proof for the existence of the NR as a distinct nuclear organelle *in vivo*. Furthermore, we substantiate the knowledge of the lamin composition of the NR by super-resolution images of the lamin A/C and B1 organization. Finally, we show that (over)expression of lamins can induce aberrant NR structures. These NR organizations *in vivo* and *in vitro* are illustrated with different types of high-resolution microscopy, including electron microscopy (EM) and Stimulated Emission Depletion (STED) microscopy.

### 1.1 Structure of the NR

**FIGURE 1:**
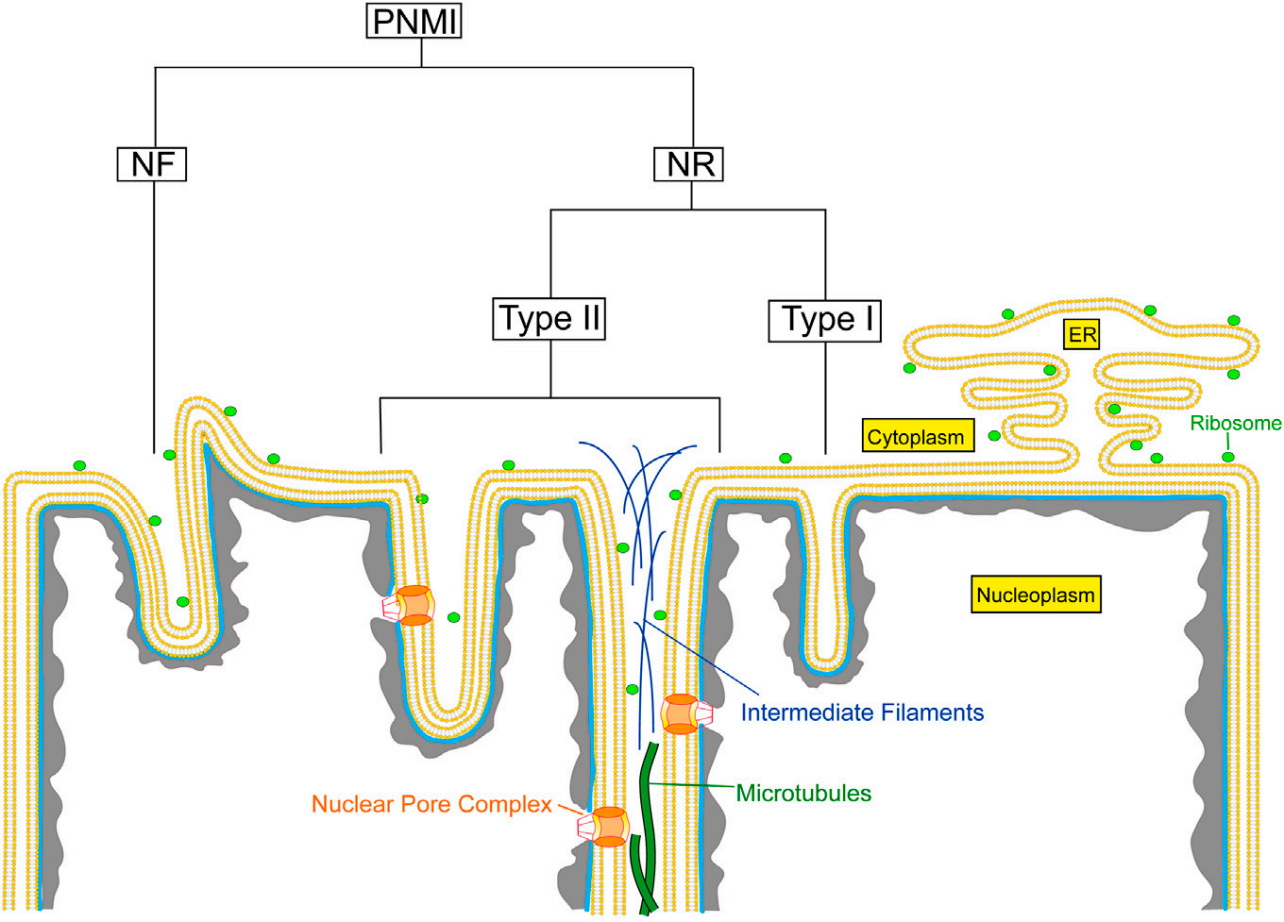
Schematic overview of the different types of nuclear membrane invaginations. PNMI, Pleiomorphic nuclear membrane invaginations; NR, Nucleoplasmic reticulum; NF, Nuclear folds.

Invaginations of the nuclear envelope occur in different shapes, sizes, and compositions. The pleomorphic appearance of these invaginations, often if not always combining the different structural entities within one nucleus, indicates that these different structures have separate functions. Mainly based on electron microscopic observations, the pleiomorphic nuclear membrane invaginations (PNMI) can be divided into the following subtypes: 1) nuclear folds (NF) resulting in surface clefts; 2) double nuclear membrane tubular invaginations of both ONM and INM, previously described as type II NR (Malhas et al., 2011); 3) invaginations of only the INM, previously described as type I NR (Malhas et al., 2011); and 4) other intranuclear structures (Figure 1). In this review, we use the term nucleoplasmic reticulum (NR) only for the previously defined type I and type II NR.

#### 1.1.1 Nuclear Folds

Within eukaryotic cells a dynamic balance exists between the folded and unfolded state of a nucleus. In 1965, folded nuclei were described for the first time. Lane (1965) demonstrated that the nuclei of smooth muscle cells disclosed clefts in their surface upon contraction. This phenomenon was suggested to occur as a consequence of mechanical forces exerted onto these nuclei during muscle cell contraction, since upon relaxation these nuclei regained their smooth contour. However, Franke and Schinko (1969) revealed that some nuclei of striated muscle cells retained their invaginations even after isolation from the muscle cells. This finding contradicted the pure mechanical explanation of nuclear folds and indicated that these nuclear folds represent a functional conformation of the nuclear membrane.

#### 1.1.2 Double Nuclear Membrane Invaginations

Fricker et al. (1997) were the first to describe in detail the complex membranous structure within the nucleus, now known as the NR. These authors described long, dynamic intranuclear tubular structures and demonstrated that these tubes consisted of a double (nuclear) membrane with enclosed nuclear pore complexes, surrounded by lamins and containing proteins such as protein disulphide isomerase and glucose-6-phosphatase. These transmembrane proteins are normally only found in the endoplasmic reticulum (ER) membrane, clearly indicating that these tubes result from invaginations of both the ONM and INM together with the underlying nuclear lamina. To date, intranuclear tubular invaginations of both the INM and ONM have been reported in a variety of cultured cells and cells in tissues, including mouse 3T3 fibroblasts (Schmidt et al., 1994; Fricker et al., 1997; Clubb and Locke, 1998), Sertoli cells (Suarez-Quian and Dym, 1992), rat small neuroblasts (Radouco-Thomas et al., 1971), raccoon and rat neurons (Burns et al., 1971; Stevens and Trogadis, 1986), human and mouse liver cells (Leduc and Wilson, 1959a, 1959b; Wier et al., 1971; Bourgeois et al., 1979), mouse skeletal muscle cells (Marius et al., 2006), and rat cardiomyocytes (Guatimosim et al., 2008). Nuclear invaginations of the INM and ONM have also been found in many tumour-derived cell lines and in virus-infected cells (see Section 1.4). Invaginations of both the INM and ONM have previously been described as type II NR (Malhas et al., 2011). According to this description, it encloses a diffusion-accessible cytoplasmic core, which often contains cytoskeletal elements. In addition, it contains nuclear pore complexes and a nuclear lamina. We emphasize that also nuclear pockets, described as cytoplasmic projections into the nucleus (Burns et al., 1971), and consisting of invaginations of both nuclear membranes (Bourgeois et al., 1979) belong to this group of NR structures. Nuclear pockets have been reported in a variety of hematopoietic cells, including leukocytes (Anderson, 1966; Smith and O’Hara, 1967b, 1967a), monocytes (Huhn, 1967), and lymphocytes (Anderson, 1966; Smith and O’Hara, 1967a, 1967b; Huhn, 1967; Sasaki and Kendall, 1985; Sasaki and Matsumura, 1988).

Before its classification as type II NR, Broers et al. (1999) further specified these double nuclear membrane invaginations by demonstrating that all three A-type lamins were found to be associated with these structures. In addition, long-term life cell imaging using GFP-tagged lamins showed that these invaginations were highly stable during interphase. Double labelling with rhodamine B hexyl ester and GFP-labelled lamins showed that all membrane-containing tubules are associated with lamins. Remarkably, Fricker et al. (1997) as well as Broers et al. (1999) also described intranuclear lamin structures (foci and tubules) without a nuclear envelope staining.

Next to tubular invaginations which seemed to be mainly vertically organized in cells growing on cover slips, more complex tangled filament networks, parallel to the cell substrate were described in cultured fibroblasts (Clubb and Locke, 1998; Broers et al., 1999). These horizontally organized tubular invaginations contained (cytoplasmic) actin fibers, while the vertical tubular invaginations lacked actin filaments (Clubb and Locke, 1998). Other studies confirmed the presence of actin in tubular invaginations (Johnson et al., 2003; Storch et al., 2007) but made no reference to the distinction between vertically and longitudinally oriented tubules.

#### 1.1.3 Single Nuclear Membrane Invaginations

In addition to type II NR, also single membrane intranuclear structures have been described, such as the annulate lamellae (AL) system (Chen and Merisko, 1988; Kessel, 1989) and the nucleolar channel system (NCS) (Clyman, 1963; Moricard and Moricard, 1964; Ancla et al., 1965; Terzakis, 1965; More and McSeveney, 1980). Invaginations of only the INM have been defined as type I NR (Malhas et al., 2011). Type I NR does not contain a cytoplasmic core, can lack nuclear pore complex components and often lacks a nuclear lamina.

AL are often abundant in cells with high proliferative capacity, such as oocytes, embryonic cells, and tumour cells, but they are also present in non-proliferating cells under permanent cell-cycle arrest, including murine neurons and cardiomyocytes (Raghunayakula et al., 2015). These cytoplasmic organelles lack a nuclear lamina, similar to the endoplasmic reticulum (Chen and Merisko, 1988), but do contain annulate pore complexes, which are morphologically comparable to nuclear pore complexes (Raghunayakula et al., 2015). AL have been proposed to function as a storage compartment for excess nucleoporins to support the assembly of NPCs during rapid cell proliferation, but this function does not seem to correspond with the AL expression in non-proliferating cells (Raghunayakula et al., 2015; Ren et al., 2019). In addition, a role for AL in viral infection is becoming evident. AL have been observed in cells infected with various viruses, including the severe acute respiratory syndrome coronavirus 2 (SARS-CoV-2) (Eymieux et al., 2021b; Caldas et al., 2021). The increase in AL might be a cellular stress reaction, but it could also be a specific induction in favour of the pathogen (Eymieux et al., 2021a). Although there are a few hypotheses, the exact biological role of AL remains largely unknown.

The NCS is an invagination of only the INM and not of the whole nuclear envelope. The NCS consists of several layers of membrane tubules embedded in an electron dense matrix and it lacks both nuclear pore complexes and a nuclear lamina, in contrast to the true tubular invaginations (Kittur et al., 2007). This structure appears transiently, during an ∼5-day window, in the midluteal phase of the menstrual cycle when the human endometrium is receptive to implantation of the fertilized egg (Kittur et al., 2007; Zapantis et al., 2013). The NCS is restricted to nuclei of endometrial epithelial cells (Clyman, 1963; Moricard and Moricard, 1964; Ancla et al., 1965; Terzakis, 1965; More and McSeveney, 1980) and is a hallmark for secretory transformation of endometrial epithelial cells. Although their exact function remains elusive, evidence points towards a role in preparing the endometrium for blastocyst attachment and implantation (Zapantis et al., 2013; Pytowski et al., 2019).

Next to these two specific structures, type I NR is also found in tumour-derived cell lines and in certain virus-infected fibroblast cells (see Section 1.4). Finally, type I NR can be induced in Schwann cells by modulating HMG CoA reductase activity (Berciano et al., 2000; Verstraeten et al., 2006).

### 1.2 Formation of the NR

The expansion and invagination of the nuclear membrane leads to the formation of the NR. Fricker et al. (1997) were amongst the first to hypothesize that the intranuclear channels result from incomplete resolution of invaginations during nuclear envelop reassembly. Normally, at the end of mitosis the invaginations are resolved by chromatin decondensation, after which the nuclear envelope vesicles fuse to from the nuclear envelope. However, if the fusion of the vesicles precedes decondensation of chromatin, it may result in a nucleus with intranuclear channels. Differential rates of decondensation of chromatin in different cell types could explain the different degree of complexity, the number of NR channels and their different orientation in particluar cell types. However, formation of new NR channels has also been reported in post-mitotic primary cells, cycle arrested cells, and during interphase in free cycling cells (Goulbourne et al., 2011; Drozdz et al., 2017). In addition, later studies showed that either alterations in the constitution of the nuclear membrane or changes in nuclear membrane associated proteins can induce formation of invaginations, thus indicating a cell cycle-independent manner of NR formation (see below).

#### 1.2.1 Mechanisms of NR Formation

Since Drozdz and Vaux (2017) described three possible mechanisms that could induce NR formation, additional evidence for all three mechanisms has been found. The first mechanism is the “pulling in” mechanism, which suggests that NR invaginations could be driven by rearrangements of chromatin tethered to the NE and pulling in the nuclear membrane. Indeed, many (dynamic) interactions between chromatin and the NE exist (Amendola and van Steensel, 2014; Ptak et al., 2021). In addition, polytene nuclei from *Drosophila melanogaster* salivary glands were found to induce NR structures after condensin-mediated chromatin compaction (Bozler et al., 2014).

Alternatively, the NR can be formed via the “pushing in” mechanism. NR formation could take place as a result of forces exerted on the NE from the outside of the nucleus (Drozdz and Vaux, 2017). These forces could be generated by the cytoskeleton, which would also explain the presence of cytoskeletal elements, such as microfilaments, intermediate filament, and microtubules, in the cytoplasmic core of type II NR (Malhas et al., 2011). Furthermore, the force could also come from the centrosomes, since the centrosomal region of granulocytic cells was found to be in close proximity to major nuclear invaginations (Olins and Olins, 2005). In addition, the microtubule network could exert forces on the nuclear envelope. In HGPS fibroblasts, a high stability of the microtubule network was found to contribute to HGPS cellular phenotypes (Larrieu et al., 2014). The aberrant nuclear morphology in HGPS patient cells, but also lamin A/C-depleted cells could be corrected by Remodelin (Larrieu et al., 2014; Larrieu et al., 2018). This small molecule targets the protein N-acetyl-transferase 10 (NAT10) and this inhibition was found to destabilize the microtubule network, thereby releasing external forces on the nuclear envelope and contributing to nuclear shape rescue. Finally, extracellular membrane vesicle (EV) loading of the endosomal compartment were recently found to induce NR structures (Section 1.3.3) (Corbeil et al., 2020). However, the “pushing in” mechanism can only explain the formation of type II NR, not type I NR.

Lastly, the NR could be assembled *de novo*, *via* a dedicated machinery. Several cellular machineries are already known to induce lipid bilayer curvature and cellular membrane invaginations, including clathrin-mediated endocytosis, clathrin-independent mechanisms, membrane curvature regulation by reticulons and DP1/YOP1, and vesicle budding by coatomer protein complex I and II (Drozdz and Vaux, 2017)*.* The variety of membrane deformation mechanisms present in cells possibly includes a selective machinery for NR formation. Additional evidence that supports *de novo* NR formation comes from studies that demonstrate that cells incorporate newly synthesized phospholipids and lamin B1 into nascent tubular NR invaginations (Drozdz et al., 2017; Pytowski et al., 2019).

It should also be noted that nuclear membranes are more elastic compared to the plasma membrane, which suggests that the nuclear membrane has an intrinsic property to fold, leading to high membrane fluctuations (Larijani et al., 2022). This elastic property likely favours the NR formation, independently of which mechanism being involved.

#### 1.2.2 The Role of Lipid Metabolism and NE-Associated Proteins in NR Formation

Several data indicate that modulating the metabolism of cholesterol greatly influences the shape of the NR (Berciano et al., 2000; Verstraeten et al., 2006). Invaginations of only the INM can be seen when cells are treated with tellurium or lovastatin (see below), both of which block the cholesterol synthesis. The mechanism leading to these additional single membrane invaginations or perhaps only membrane lobulations is unclear. Possibly, the absence of membrane stabilization by cholesterol causes local membrane extensions without formation of nuclear pore complexes (NPC).

An essential enzyme in the process of NR formation is the CTP:phosphocholine cytidylyltransferase (CCTα) enzyme. CCTα is a rate-limiting enzyme in the choline phosphotransferase (CTP)-choline pathway for phosphatidylcholine (PC) synthesis. Upon activation by fatty acids, the enzyme translocates to the NR and stimulates the proliferation of the NR (Lagace and Ridgway, 2005). The activity and membrane-binding function of this enzyme is essential for the formation of the NR, as the activation of the enzyme with oleate or enhancing its membrane binding capacity, leads to an increase of NR formation (Lagace and Ridgway, 2005; Gehrig et al., 2008). Accordingly, the expression of CCTα-GFP mutants with a compromised catalytic or membrane binding affinity decreases induced NR formation. On the other hand, oleate treatment of CHO-K1 cells resulted in an increase of NR structures within 2 h (Lagace and Ridgway, 2005; Gehrig et al., 2008).

The altered expression of CaaX motif containing lamins (see Section 1.2.3) or other proteins associated with the INM, such as lamin B receptor (LBR) (Ellenberg et al., 1997; Ma et al., 2007), certain mutants of fibroblast growth factor receptor 4 (Sorensen et al., 2004), mouse germ cell-less protein (Kimura et al., 2003), nucleoporin 153 (Bastos et al., 1996; Marelli et al., 2001), or Nopp140 (Isaac et al., 2001), leads to the expansion of the nuclear membrane and the formation of intranuclear membrane structures. Different mechanisms have been described to explain the formation of NE invaginations as a result of altered INM protein expression. For LBR, the transmembrane segment is involved in nuclear membrane overproduction, while the N-terminus interacts with chromatin or chromatin-associated proteins, leading to NE invaginations (Ma et al., 2007). An explanation for the formation of nuclear invaginations by Nopp140, often described as R-rings, is based on the interaction of its highly charged repeat domain with the head groups of phospholipids. The positively charged repeats can bind directly to the head groups of phospholipids, while the negatively charged repeats can interact with phospholipids via calcium bridges (Isaac et al., 2001; Kittur et al., 2007).

#### 1.2.3 The Role of Lamins in NR Formation

Next to the association of the NR with several organelles, type II NR also contain a nucleoplasmic lamina.

Nuclear lamins are thought to have a wide variety of functions, which can also be expected from the many interactions they have with the INM and within the nucleoplasm. Lamins have more than a hundred lamin-binding proteins, most of which are integral proteins of the INM (Gruenbaum and Medalia, 2015). The functions assigned to the lamins include playing a role in chromatin organization (Galiová et al., 2008; Dechat et al., 2009), mitosis (Dittmer and Misteli, 2011; Naetar et al., 2017), and apoptosis (Rao et al., 1996). Furthermore, lamins are involved in providing structural support to the cells and in mechanosensing and mechanoresponse of cells (Stiekema et al., 2020). Also the lamin in the NR structures is important in maintaining the nuclear architecture (Section 1.3.1).

**FIGURE 2:**
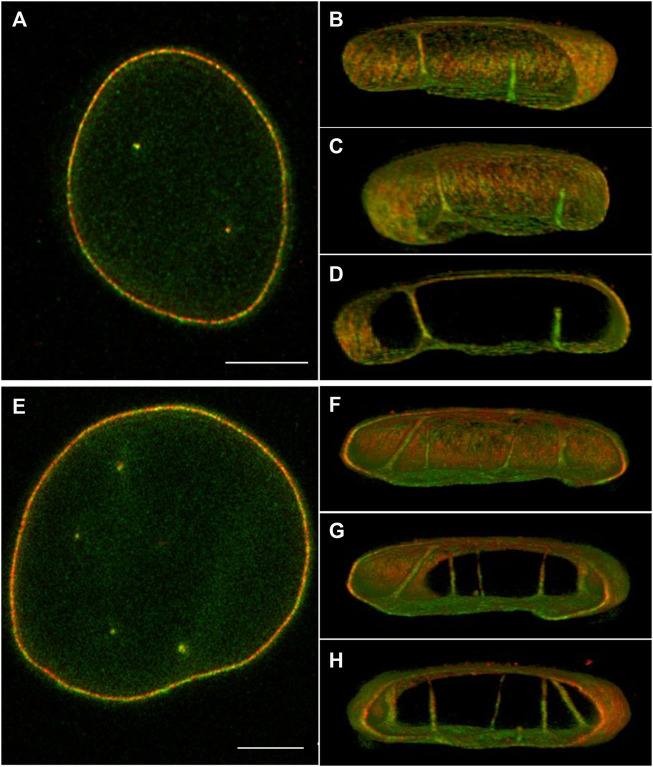
3D STED of normal human dermal fibroblast cells, stained for lamin A/C (red) and B1 (green). **(A,E)** 2D image of the middle of the nucleus. **(B–D)** Different 3D reconstruction views of the cell in **(A)** using ImageJ 3D viewer. **(F–H)** Different 3D reconstruction views of the cell in **(E)** using ImageJ 3D viewer. Scale bars indicate 5 μm. Also see Supplementary Figures S1,S2. Cell culture and immunostaining of NHDF was performed as described previously (Stiekema et al., 2021). STED images were taken with an abberior Instruments INFINITY LINE microscope equipped with an inverted IX83 microscope (Olympus), a 60× oil objective (UPlanXApo 60×/1.42 oil, Olympus), using pulsed excitation lasers at 561 nm (for secondary antibody Abberior STAR ORANGE) and 640 nm (for Abberior STAR RED) and a pulsed STED laser operating at 775 nm. All acquisition operations were controlled by the Lightbox Software. STED images were deconvoluted as described in Stiekema et al. (2021).

To gain more insight into the distribution of lamins A/C and B1 in the NR, we imaged cultured normal human dermal fibroblasts (nHDF), immunostained for lamin A/C and B1 with three-dimensional high resolution STED microscopy (Figure 2; Supplementary Figures S1,S2). These images clearly demonstrate that both lamin A/C and lamin B1 are associated with NR tubules, as also shown by 3D Stochastic Optical Reconstruction Microscopy (3D-STORM) (Schoen et al., 2017). Drozdz and Vaux (2017) also recently visualised lamins in NR tubules in nHDF with super resolution light microscopy, although these seem to mainly consist out of lamin B1. Some tubules transverse the entire nucleus, while others are (much) shorter in length and end in the nucleoplasm. In addition, from these fluorescence images it became evident that the thickness of the lamina layer associated with the tubules varies.

The interaction of CCTα with nuclear lamins appears to be important for NR formation (Gehrig et al., 2008). The presence (Prufert et al., 2004) and the proper processing (Maske et al., 2003) of the CaaX-motif in lamins is crucial for the incorporation of lamins into the nuclear lamina and their association with the nuclear membrane. The processing of lamins, except for lamin C, consists of the farnesylation of the CaaX-motif, proteolytic cleavage of the last three COOH-terminal amino acid residues of the CaaX motif, and carboxymethylation of the farnesylated cysteine. Maturation of lamin A requires a second cleavage that removes 15 amino acids from the C-terminus, along with the farnesylated and carboxymethylated residues (Simon and Wilson, 2013; Tatli and Medalia, 2018). Modulation of lamin farnesylation also effects the NR formation. This becomes apparent when treating cells with farnesyltransferase-inhibitors or statins, the latter blocking cholesterol metabolism and as a result the farnesylation of, amongst others, lamins. As a result, non-farnesylated prelamin accumulates and the number and size of tubular invaginations of the nuclear envelope increases (McClintock et al., 2006; Verstraeten et al., 2006). Accumulation of farnesylated prelamin using non-peptidomimetic compound N-acetyl-S-farnesyl-L-cysteine methylester (AFCMe) leads to an accumulation in the nucleoplasm, resulting in the formation of even more tubular invaginations (Lattanzi et al., 2007; Mattioli et al., 2008). Taken together, both prenylated and not-prenylated lamin A causes NR formation, but the permanent attachment of a farnesyl group to lamin A has a more dramatic effect on the induction of the NR formation.

Overexpression of lamins with the CaaX motif (lamins A, AΔ10, B1, B2, and Dm0) leads to proliferation of the nuclear envelope, resulting in lobulated nuclei with highly folded nuclear membranes and invaginations of the nuclear membrane (Broers et al., 1999; Prufert et al., 2004).

**FIGURE 3:**
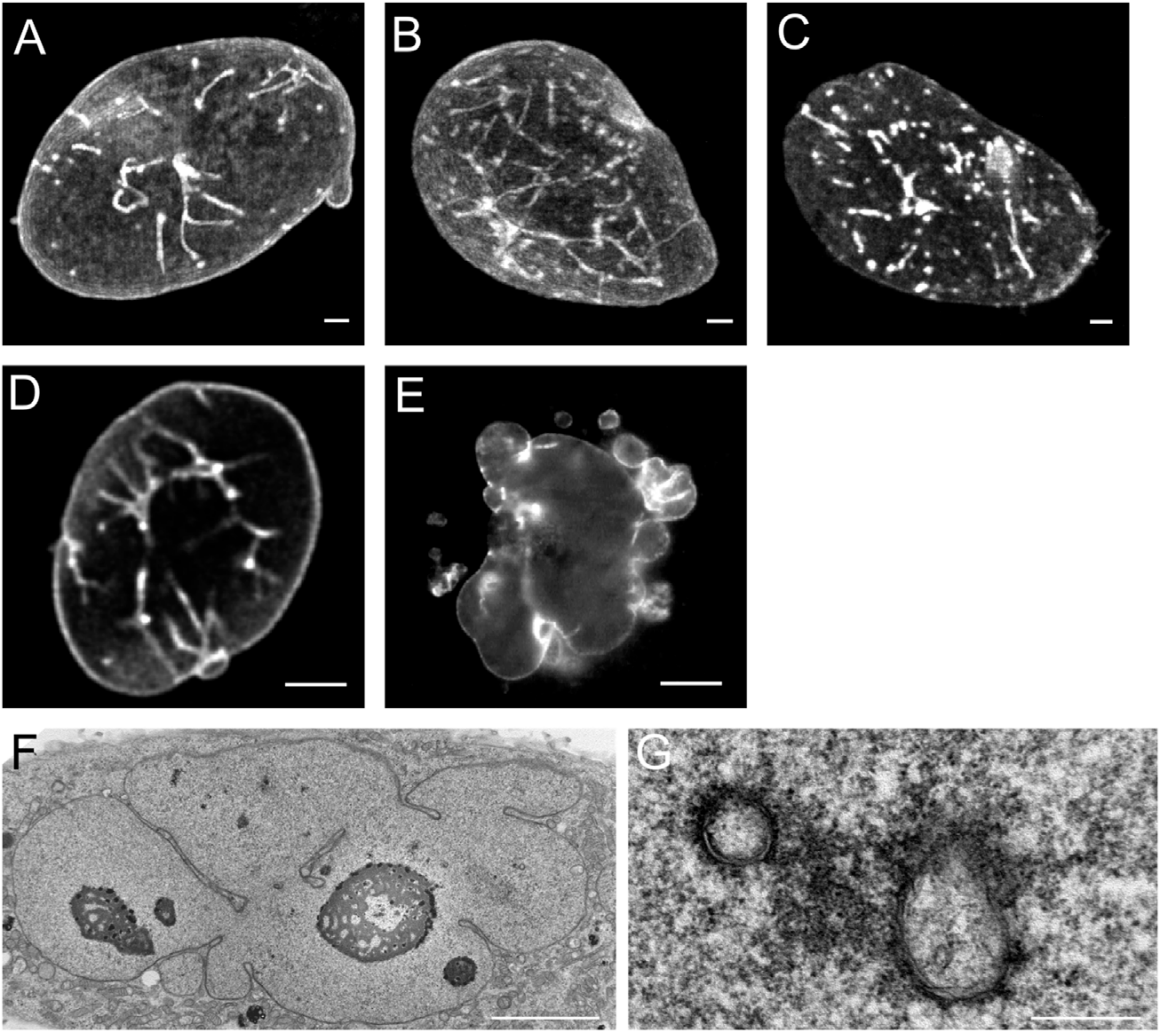
Effects of over-expression of different types of lamins on the nucleus of CHO-cells. **(A)** The over-expression of lamin A leads to nuclear membrane growth and the formation of a large number of tubular invaginations of the nuclear envelope. Scale bar indicates 10 μm. **(B)** The over-expression of lamin AΔ10 leads to nuclear membrane growth and the formation of a large number of tubular invaginations of the nuclear envelope. Scale bar indicates 10 μm. **(C)** The over-expression of lamin C leads to mainly aggregates in the nucleoplasm. Scale bar indicates 10 μm. **(D)** The over-expression of lamin B1 leads to nuclear membrane growth and the formation of a limited number of tubular invaginations of the nuclear envelope. Scale bar indicates 10 μm. **(E)** The over-expression of lamin B2 leads to enlarged nuclei with highly folded nuclear membranes and lobulations of the nuclear membrane. Scale bar indicates 10 μm. **(F)** EM-recording of tubular invaginations in lamin A over-expressing CHO cells. Scale bar indicates 5 μm. **(G)** Detailed recording of tubular invaginations in lamin A over-expressing CHO cells. Scale bar indicates 0.5 μm. CHO-cells were grown, transfected and imaged as described (Broers et al., 1999). Confocal fluorescence images (A‐E) show maximal Z-stack projections.

Figure 3 illustrates the specific effects of over-expression of different subtypes of lamins in CHO-cells. Over-expression of lamin A-GFP and lamin AΔ10-GFP (Figures 3A,B) resulted in increased tubule formation but not into a prominent increase in lobulation of the nuclei. Further examination of CHO cells over-expressing lamin A by electron microscopy clearly demonstrates that these invaginations and tubes consist of a double membrane (Figures 3F,G). Upon over-expression of lamin C, not containing the CaaX-motif, aggregates inside the interior of the nucleus occur without growth of the nuclear membrane or additional tubule formation (Figure 3C, see also Broers et al., 1999; Prufert et al., 2004). Transfection with lamin B1-GFP (Figure 3D) resulted in nuclear lobulation and a moderate increase in tubular invaginations. Depending on the length of the lamin B2-GFP construct, different effects can be seen. Over-expression of lamin B2-GFP lacking the first 20 amino acids resulted in dramatic lobulation of the nuclear envelope (Figure 3E, see also Schumacher et al., 2006), with only a slight increase in true intranuclear tubule formations. Over-expression of the complete lamin B2-GFP construct did not lead to lobulation of the nucleus (Schumacher et al., 2006).

### 1.3 Functions of the NR

The occurrence of variable amounts of NR structures in different cell types and the finding of several studies that the amount of NR structures can be regulated both *in vivo* and *in vitro*, clearly proves that the NR is a functional unit of the nucleus (Section 1.1). In addition, the formation of the NR appears to be a complex process with a lot of different proteins involved, which also suggests that it is involved in numerous nuclear functions (Section 1.2). In the following paragraphs we discuss the many theories about the exact function of the NR that were formulated over the years, which are not mutually exclusive.

#### 1.3.1 Role of the NR in Maintaining the Architecture of the Nucleus

Several high-resolution imaging studies investigating the tubular invaginations of the NR have been performed *in vitro*, with a variety of cell cultures. An early three-dimensional electron microscopy reconstruction of these tubular invaginations in PC12 cells has been published by Stevens and Trogadis (1986). Nuclear clefts and folds were first hypothesized to be a consequence of mechanical forces exerted on cells (Lane, 1965). This report on clefts and folds in nuclei of smooth muscle cells during contraction forwarded the hypothesis that the nuclei change their shape to cope with the mechanical stress during compression and relaxation, eventually protecting the integrity of the nucleus and the whole cell (Lane, 1965).

**FIGURE 4:**
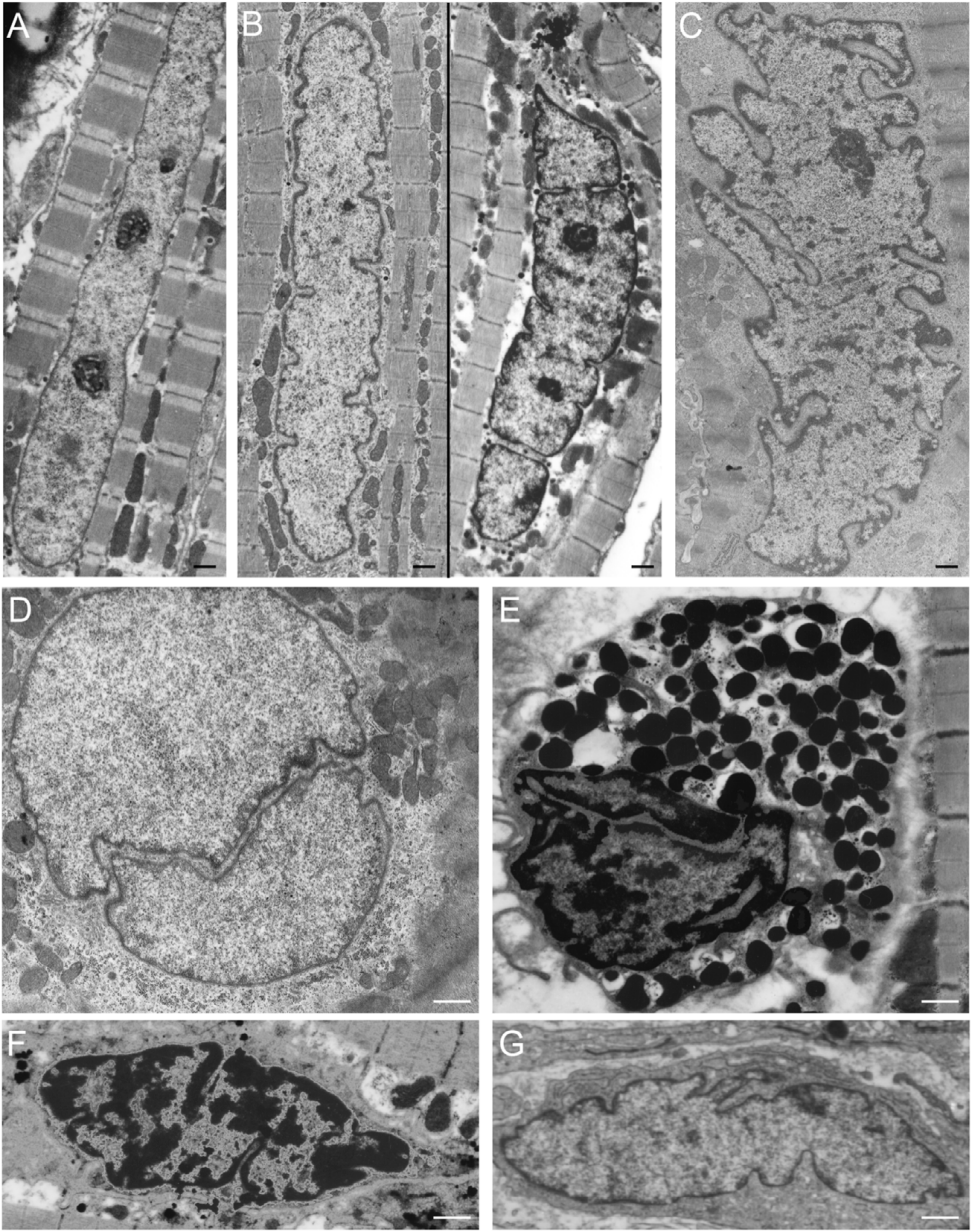
NR-like invaginations of the nuclear envelope in different cardiovascular cell types as seen at the electron microscopy level. **(A)** Cardiomyocyte in relaxation (goat *in vivo* atrial tissue), the nuclear membrane shows no invaginations or folds. **(B)** Cardiomyocyte in mild contraction (goat *in vivo* atrial tissue), the nuclear membrane shows some folds. **(C)** Cardiomyocyte in strong contraction (goat *in vivo* atrial tissue), the nuclear membrane is strongly folded. **(D)** Cardiomyocyte in relaxation (goat *in vivo* atrial tissue) demonstrating a large invagination of the nuclear envelope. **(E)** Mast cell in cardiac tissue (human ventricle), the nuclear membrane has large invaginations. **(F)** Capillary endothelial cell in cardiac tissue (human ventricle) with several invaginations of the nuclear envelope. **(G)** Fibroblast in cardiac tissue (human ventricle), the nuclear membrane is strongly folded. Scale bars indicate 1 μm. Goat atrial samples were fixed for 2 h in 3% glutaraldehyde buffered to pH 7.4 with 90 mM KH_2_PO_4_. Thereafter the samples were washed in the same buffer for 24 h and post-fixed for 1 h in 2% OsO_4_ buffered to pH 7.4 with veronal acetate. Next, the samples were rapidly dehydrated through a graded series of ethanol and routinely embedded in Epon (Driesen et al., 2009). Ultra-thin sections were counterstained with uranyl acetate and lead citrate prior to examination in a Philips CM 100 electron microscope.

Furthermore, *in vivo* data confirm the presence of nuclear clefts in cardiomyocytes, which are continuously exposed to force differences (Figures 4A–C). With a stronger contraction the abundance of nuclear invaginations and folds increases in cardiomyocytes (Figures 4A–C). This finding suggests an increasing need for these structural alterations with higher mechanical forces. In addition, increasing foldings, convolutions, and intranuclear tubules were reported in patients with cardiac hypertrophy by Ferrans et al. (1975) and hypothesized to be a response to the stimulus of hypertrophy. In contrast, cardiomyocytes in relaxation can either lack or still contain nuclear invaginations or folds (Figure 4D). Similarly, nuclear invaginations are also visible in mast cells, capillary endothelial cells, and fibroblasts in cardiac tissue (Figures 4E–G). The presence of the nuclear invaginations and folds in cells that experience no mechanical forces indicates a structural function of these structures. This does not rule out the possibility of a flexible regulation of the nuclear folds that facilitates coping with increasing mechanical forces by upregulating the number of nuclear folds. The finding that comparable clefts and folds also occur in nuclei of non-contracting isolated muscle cells, points toward a more structural and less dynamic adaptation of nuclei to stress. In other cell types, Lammerding et al. (2004) and Broers et al. (2004) reported that nuclei have a coping strategy to withstand mechanical compression, and that A-type lamins play an essential role in this process. Indeed, the tubular invaginations could provide structural support during mechanical compression (Broers et al., 1999). The vertical orientation and stability of the observed tubules points to a role in structural support of the nucleus in cell cultures.

#### 1.3.2 Function of the NR in Nucleocytoplasmic Transport

Bourgeois et al. (1979) hypothesized that the nuclear tubular invaginations of the nuclear membrane in kidney, liver, and carcinoma cells have a functional role in nucleo-cytoplasmic transport, as they form associations with nucleoli. Other groups also demonstrated a (partial) association of the nuclear tubular invaginations with nucleoli (Dupuy-Coin et al., 1986; Fricker et al., 1997; Clubb and Locke, 1998; Abe et al., 2004; Lagace and Ridgway, 2005; Lee et al., 2006). The distance between the deeply buried nucleoli and the nuclear envelope is largely reduced by the presence of a tubular invagination, thus facilitating the transport of mRNA out of the nucleus. As Fricker and collaborators stated in 1997 (Fricker et al., 1997), even a single tube traversing the nucleus decreases the maximal distance between a random location in the nucleoplasm and the nuclear envelope with 50%. With only a few tubes, the distance of any location of the nucleoplasm and nucleolus to the nuclear envelope is reduced to less than 0.5 μm. In general, the expansion of the interface-surface between the nucleus and the cytoplasm will facilitate the communication and transport between the two compartments (Gehrig et al., 2008).

The NR was recently also found to be involved in the intracellular transfer pathway of extracellular vesicles (EVs) (Santos et al., 2018). EVs, such as exosomes and microvesicles, are nano-biological membrane structures important in cell-to-cell communication and their actions can lead to favoring proliferation versus differentiation of stem cells, inducing epithelial-mesenchymal transition, and modulating immune responses. However, EVs are also involved in pathological conditions, such as in developing a pre-metastatic niche in cancer (Corbeil et al., 2020). Recently, late endosomes associated with the NR and nuclear localization of EV-derived proteins was observed in cancer cells, mesenchymal stromal cells in cultures, and in breast cancer patient biopsies (Rappa et al., 2017; Santos et al., 2018). Rappa et al. (2017) reported that the proportion of cells with NR-associated late endosomes increased and the proportion of cells without NR structures decreased upon exposure to EVs. This observation might also be an indication that EV-loading of the endosomal compartment regulates NR-biogenesis, thereby supporting the “pushing in” mechanism of NR formation. Santos et al. (2018) reported that the VOR-complex, composed of vesicle-associated membrane protein-associated protein A (VAP-A), oxysterol-binding protein-related protein 3 (ORP3), and the small GTPase Rab7, is essential for the localization of the late endosomes in the NR and the nuclear transfer of the ER-derived components. As a result of these findings, the VOR complex may become a novel drug target to impair the intercellular communication in the cancer microenvironment. Indeed, itraconazole treatment disrupts the VOR complex and inhibits EV-mediated pro-metastatic morphological changes and migratory properties of colon cancer cells (Santos et al., 2021).

#### 1.3.3 Function of the NR in Signalling

An early hypothesis by Franke and Schinko (1969) stipulated that the invaginated shape of the nucleus was induced by intracellular changes in ion concentration. More recently, however, evidence has been forwarded for the reverse phenomenon, i.e., that the invaginations of the nucleus function as regulators of intra-nuclear ion concentrations.

Since the NR is in a continuum with the luminal space of the ER, it can function as a calcium store (Somlyo et al., 1985; Gerasimenko et al., 1995; Lui et al., 1998a; Lui et al., 1998b; Echevarría et al., 2003; Lui et al., 2003; Marius et al., 2006). Upon stimulation, calcium can be quickly released into the nucleoplasm from the nuclear envelope and the NR. Like the nuclear membrane, the NR also contains IP3-receptors (Echevarría et al., 2003; Lui et al., 2003; Guatimosim et al., 2008) and ryanodine-receptors (Marius et al., 2006; George et al., 2007; Guatimosim et al., 2008), indicating that these invaginations are functional in delivering calcium from the NR into the nucleoplasm. Because of the structural organization of the NR, calcium can easily be released into all regions of the nucleoplasm and induces a fast overall response throughout the whole nucleoplasm. As such, this is a potential mechanism for a differentially regulated calcium metabolism in distinct sub-compartments of the nucleoplasm (Phair and Misteli, 2000). An indication of a differentiated response in each of the sub-compartments of the nucleus is given by the non-homogenous occurrence of the IP3 receptor and ryanodine-receptor in the NR (Lui et al., 2003; Marius et al., 2006; George et al., 2007; Guatimosim et al., 2008) and by the dynamic behavior of the NR (Lui et al., 1998a; Lee et al., 2006).

Calcium in the nucleus plays an important role in the process of apoptosis (Nicotera and Rossi, 1994) and the shuttling of proteins across the nuclear envelope (Stehno-Bittel et al., 1995; Perez-Terzic et al., 1996). Also, gene transcription through the activation of cAMP response elements (Hardingham et al., 1997; Chawla et al., 1998), Elk-1 (Pusl et al., 2002) or the direct binding of calcium to nuclear transcription factors (Dobi and Agoston, 1998), is based on changes in intra-nuclear calcium concentration. However, the necessity of a NR as a functional calcium store is to be cell- and tissue-specific, as in neurons nuclear calcium signals can be independent of the presence of a NR in neurons (Bezin et al., 2008).

The concept of the NR playing a role in signaling pathways is also supported by data of Storch et al. (2007), who hypothesized that the α smooth muscle actin (α-SMA) filaments found in tubular invaginations in the nucleus is essential in mechanotransduction during mechanical stress, because α-SMA is localized closely to the ONM of the tubular invaginations and is continuous with the cytoplasmic actin filaments.

#### 1.3.4 Function of the NR in Cell Differentiation

The role of the NR in cell differentiation has largely been deduced from the large variety in the frequency of invaginations in differentiated versus undifferentiated cells (Johnson et al., 2003). Apparently, undifferentiated or cancerous cells (Section 1.4) contain a more extensive NR than fully differentiated cells. Johnson and coworkers (Johnson et al., 2003) revealed that the MDA-MB-231 cell line, a very aggressive, highly dedifferentiated human mammary epithelial tumor cell line, contains much more cells with nuclear invaginations (90%) compared to other cell line types such as of non-transformed (MCF-10A: 25%) or mildly dedifferentiated human mammary epithelial cells (MCF-7: 9%), colorectal epithelial cells (SW-480: 14% cells with invaginations), or fibroblast cells (NIH-3T3: 3% and WI-38: 9%). A possible explanation for this phenomenon could be that highly proliferative cells need more nucleo-cytoplasmic transport for exchange of mRNA and proteins in and out of the nucleus. As described above, the NR provides the extranuclear envelope surface that is necessary for such additional nucleo-cytoplasmic transport.

**FIGURE 5:**
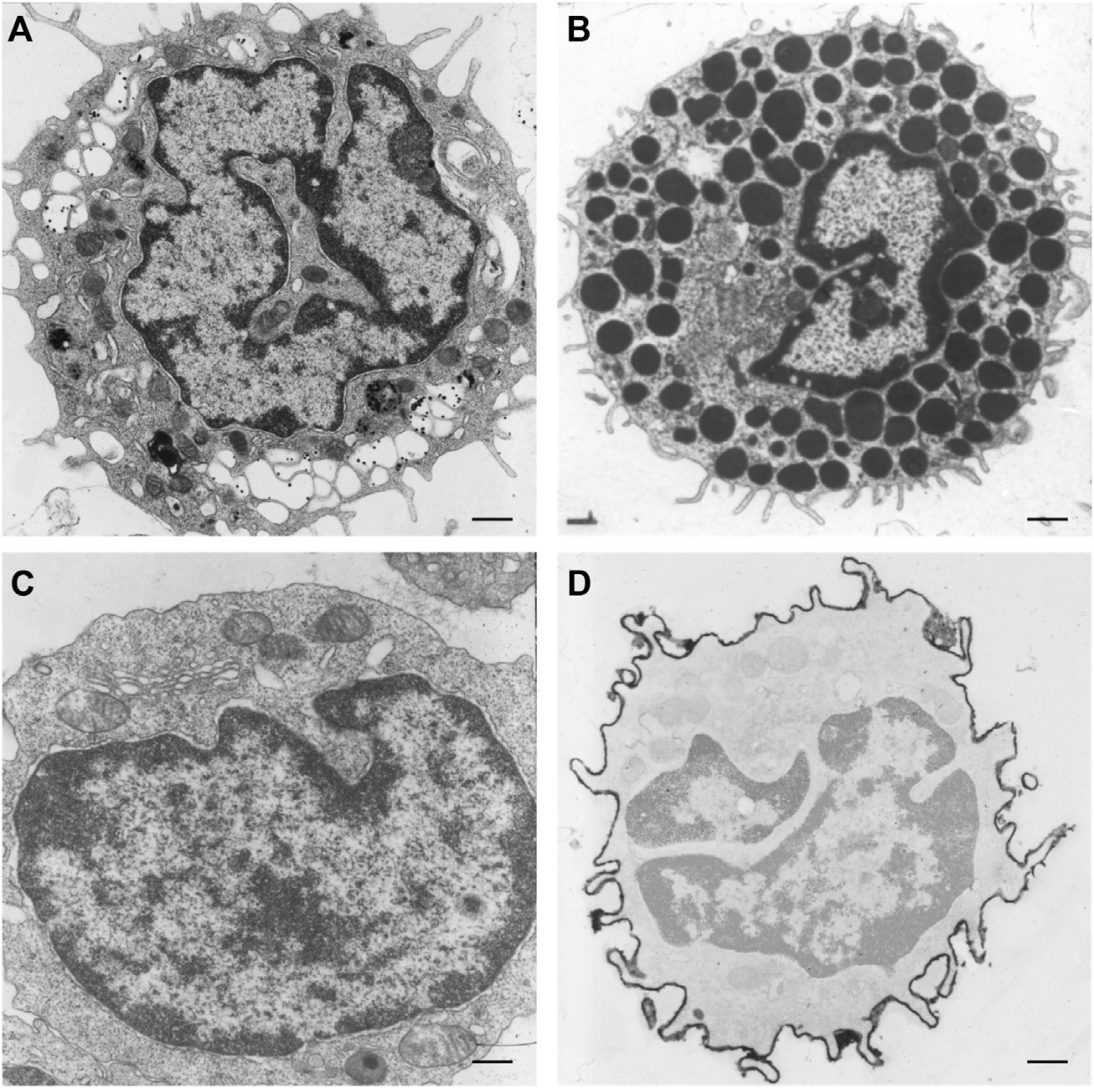
NR invaginations in human hematopoietic cells at the electron microscopy level. **(A)** Macrophage. **(B)** Mast cell. **(C)** T-lymphocyte. **(D)** B-lymphocyte. Scale bars indicate 1 μm. Human hematopoietic cells were cultured for at least 48 h in a culture dish after which the medium was discarded. Next, the cells were shortly rinsed with PBS and subsequently fixed at room temperature with 3% glutaraldehyde in 0.1 M cacodylate buffer (pH 7.4) supplemented with 0.5 mM CaCl_2_ for at least 24 h. The cells were then postfixed with 2% OsO_4_ in cacodylate buffer (pH 7.4) containing 1.5% potassium ferricyanide at 48°C for 1 h. After a short rinse in cacodylate buffer, the cells were further dehydrated in graded ethanol series before embedding in epon. Ultra-thin sections were counterstained with uranyl acetate and lead citrate prior to examination in a Philips CM 100 electron microscope.

Differences in the organization and extent of the NR in relation to the state of differentiation are clearly visible when studying electron microscopy images of hematopoietic cells *in vivo*. Macrophages, mast cells, T-lymphocytes, and B-lymphocytes, show different organizations of these nuclear pockets consisting of type II NR structures (Figure 5). This diversity of nuclear invaginations likely reflects their functionality, as Skinner and Johnson (2017) described that the diverse morphology of nuclei has clear functional impacts. The theory that the NR functions in cell differentiation is also supported by the finding of a specific type of nuclear membrane invaginations in HL-60 myeloid leukemia cells. Olins et al. (1998), Olins and Olins (2009) reported nuclear envelope-limited chromatin sheets which are invaginations consisting of sheets of chromatin covered at both sides with INM (although the presence of type I NR is not excluded). These structures are considered to play a role in cell differentiation, although their function is still to be solved.

Finally, a specific NR organization is observed during the differentiation stages of mono-nuclear Hodgkin cells to multi-nucleated Reed-Sternberg cells in classical Hodgkin’s lymphoma (Contu et al., 2018; Bienz et al., 2020). Impressive lamin A/C intranuclear septa are seen in bi- to multi-nucleated Reed-Sternberg (RS) cells, where lamin staining showed internal lamin A/C structures, subdividing the nuclei into two or more smaller compartments. These results show that this specific lamin A/C spatial organization may be instrumental in the transition from mononuclear Hodgkin to bi- and multi-nucleated Reed-Sternberg cells. Contu et al. (2018) therefore concluded that the technology of 3D analysis of lamins in classical Hodgkin’s lymphoma holds the potential for becoming a predictive tool in the clinical management of this complex disease.

#### 1.3.5 Function of the NR in Other Cellular Processes

Next to the role of the NR in providing structural support for the nucleus, nucleocytoplasmic transport, calcium signalling, and cell differentiation, evidence points towards a role in transcription, DNA repair, and lipid metabolism.

Microscopic co-localization studies have often associated NR invaginations with sites of active transcription of ribosomal genes, including nucleoli. Combined with the NPCs and cytoplasmic core in type II NR this suggests a role for the NR in facilitating nuclear export of rRNA (Drozdz and Vaux, 2017). In addition, the NR has been found to be closely associated with several repressive complexes and heterochromatin, implying a role of the NR in chromatin organization and transcription regulation (Legartová et al., 2014; Jorgens et al., 2017). Next, histone-deacetylase inhibitor trichostatin A cell treatment induced a higher NR abundance, in support of a role of the NR in general RNA export and increased gene expression (Galiová et al., 2008). Furthermore, the NR is associated with DNA lesions induced by γ-radiation, indicating a role in DNA repair (Legartová et al., 2014). The NR also might be involved in the exclusion of extrachromosomal circular DNA (eccDNA), which mostly arises from non-coding DNA during DNA damage repair. In young and healthy cells, the eccDNA relocalizes to the NPCs along nuclear actin and is excluded from the nucleus through functional NPCs. In aging and age-related disorders, eccDNA exclusion declines, which could be explained by amongst others a reduction of functional NPCs and deregulated nuclear actin. As the NR shortens the distance between the nucleolus and the nearest NPC, it potentially facilitates the exclusion of eccDNA from rDNA within the nucleolus (Qiu et al., 2021).

Another cellular process that is associated with the NR, is lipid metabolism. Type I NR has been found to associate with luminal lipid droplets (LDs) in hepatocytes (Ohsaki et al., 2016). These NR-associated luminal LDs accumulate as a result of ER stress and turn into nucleoplasmic LDs by escaping into the nucleoplasm. The molecular mechanism related to this is likely associated with the lamin deficiency in type I NR, which make the membrane weak and prone to disruption when the luminal LDs grow larger (Sołtysik et al., 2019; Fujimoto, 2022). The nucleoplasmic LDs recruit CCTα, which is pivotal in PC synthesis, as described above. These findings suggest a role of the NR in lipid metabolism, specifically to regulate PC synthesis in accordance with ER stress levels (Sołtysik et al., 2019).

### 1.4 The NR in Disease

**FIGURE 6:**
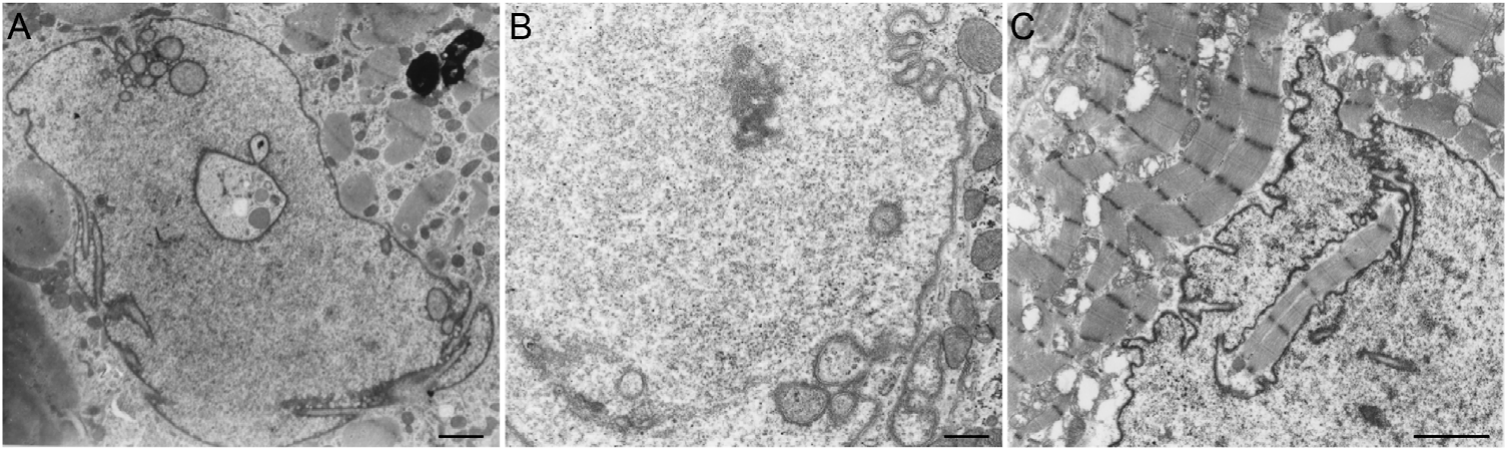
**(A)** Cardiomyocyte (human ventricular hibernating myocardium; reversible heart failure *in vivo*) demonstrating tubular invaginations of the nuclear envelope. Scale bar indicates 5 μm. **(B)** Cardiomyocyte nucleus (human ventricular hibernating myocardium; reversible heart failure *in vivo*) demonstrating tubular invaginations of the nuclear envelope. Scale bar indicates 2 μm. **(C)** Cardiomyocyte (human ventricular myocardium from dilated cardiomyopathy) demonstrating a large invagination of the nuclear envelope, engulfing contractile elements. Scale bar indicates 2 μm. Samples were prepared as described for Figure 4.

Several pathologies, such as laminopathies, cancer, (reversible) heart failure, and Alzheimer’s disease are associated with an altered NR abundance. Strikingly, in these conditions the amount of A-type lamins throughout the nucleus is generally decreased (Ausma et al., 1996; Moss et al., 1999; Wu et al., 2009) or the organization of lamins or lamin-associated proteins is disturbed (Gonzalez-Alegre, 2004). Next to cells with ill-processed lamins, cardiac cells from hibernating myocardium (reversible heart failure) regularly demonstrate tubular invaginations of the nuclear envelope, occasionally engulfing contractile elements (Figure 6).

Laminopathies are rare diseases, mainly caused by mutations in the A-type lamin gene (*LMNA*). In cells with these mutations a variety of nuclear abnormalities can be observed. These include nuclear herniations, honeycomb-like structures, and even donut-shaped nuclei. In addition, in some laminopathies, a dramatic increase of NR structures can be observed. The farnesylation-status of the CaaX-motif of prelamin A plays an important role in the appearance of invaginations of the nuclear envelope (McClintock et al., 2006; Verstraeten et al., 2006). When farnesylated prelamin A accumulates, as in restrictive dermopathy (Navarro et al., 2004, Navarro et al., 2005; Verstraeten et al., 2006), or when mutant farnesylated lamin accumulates, as in Hutchinson-Gilford Progeria Syndrome (HGPS) (McClintock et al., 2006), more tubular invaginations are formed compared to cells of healthy individuals. Also, naturally ageing cells contain more progerin/prelamin A (Caron et al., 2007; McClintock et al., 2007), and demonstrate a more abundant NR (McClintock et al., 2007) compared to cells of young individuals. Lamins can be isoprenylated through an alternative pathway, using geranylgeranyltransferase (Kuipers and van den Elsen, 2007). When HGPS cells are treated with both a farnesyltransferase inhibitor and a geranylgeranyltransferase I inhibitor, the number of nuclear abnormalities and tubular invaginations decline drastically (Varela et al., 2008). These findings confirm the importance of lamin farnesylation in NR formation. However, also other lamin A mutants have been shown to increase the NR tubule abundance (Drozdz and Vaux, 2017).

Recently, Alzheimer’s disease and associated tauopathies were suggested to be acquired neurodegenerative laminopathies because of the relation between B-type lamin dysfunction in mediating neuronal death. Studies by Frost (2016), Frost et al. (2016) reported an expansion of the NR and reduction of lamin B1 levels in neurons of tau transgenic *Drosophila* and postmortem human Alzheimer’s disease brains. Dysfunction of B-type lamins in adult neurons impact heterochromatin formation, cell cycle activation, and neuronal survival. Furthermore, an accumulation of polyadenylated RNAs within and adjacent to tau-induced NR has been reported, possibly driven by lamin dysfunction and NR formation (Cornelison et al., 2019). The exact functional consequences of the NR in tauopathies warrants further investigation, including the effect on nuclear calcium signaling, which is of high importance for synaptic activity in neurons.

As already briefly described, in cancer both type I and II NR are observed. Tumor cells in which type II NR could be observed, sometimes in combination with type I NR, include Ehrlich ascites tumor cells (Wessel and Bernhard, 1957; Bernhard, 1958; Yasuzumi and Sugihara, 1965; Burns et al., 1971), Yoshida (Locker et al., 1968) and Novikoff hepatoma cells (Babai et al., 1969), human oviduct tumor cells (Bourgeois et al., 1979), human epidermoid carcinoma cells (Bourgeois et al., 1979), neoplastic-Burkitt tumor cells (Epstein and Achong, 1965), leukemic leukocytes (McDuffie, 1967; Schuurmans Stekhoven and Holland, 1986), splenic lymphosarcoma ascites tumor cells (Levine et al., 1968), pancreatic carcinoma cells (Kamei, 1994), and breast cancer cells (Bussolati et al., 2007).

In ascites AH 602 hepatoma cells (Hoshino, 1961), Ehrlich ascites tumor cells (Yasuzumi and Sugihara, 1965) and Novikoff hepatoma cells (Babai et al., 1969; Karasaki, 1970), type I NR, in combination with type II NR, were found to form thin canaliculi. These invaginations of the INM are covered with a layer of heterochromatin, and the lumen is an extension of the perinuclear space, and not of the cytoplasm like in double membrane invaginations (Hoshino, 1961; Yasuzumi and Sugihara, 1965; Babai et al., 1969; Karasaki, 1970).

Viruses that induce type II NR include parapox viruses stomatitis papulosa and orf (Pospischil and Bachmann, 1980) and herpes simplex virus (Stannard et al., 1996). After the infection with the virus, the type II NR occurs, although also type I NR is described after virus infections (Severi et al., 1988; Nassiri et al., 1998; Buser et al., 2007; Camozzi et al., 2008; Hamirally et al., 2009). This induction of a higher amount of NR after virus infections can be caused by lamin reorganization, since virus infection causes the reorganization of the nuclear lamina through either phosphorylation or dephosphorylation of lamins (Radsak et al., 1991; Scott and O’Hare, 2001; Muranyi et al., 2002; Reynolds et al., 2004; Marschall et al., 2005; Park and Baines, 2006; Leach et al., 2007; Morris et al., 2007; Mou et al., 2007; Camozzi et al., 2008; Lee et al., 2008; Hamirally et al., 2009; Stiekema et al., 2020). This reorganization leads to an uneven thickness of the nuclear lamina, possibly leading to lobulation of the nuclear membrane and the formation of invaginations of the INM (Reczko and Boegel, 1962; Anisimová et al., 1973; Bachmann et al., 1979; Pospischil and Bachmann, 1980; Stannard et al., 1996; Braunagel et al., 1998; Hamirally et al., 2009). Villinger et al. (2015) even described extremely complex type I NR in HCMV infected cells and categorized the INM invaginations in first, second and third order infoldings. First order infoldings would be formed following initial lamin disruption. Next, second order infoldings could originate by invaginations into first order infoldings. Finally, third order infoldings could be form by invaginations of perinuclear space into second order infoldings. Also, the accumulation of specific viral proteins, together with modulated lamin proteins, can initiate the formation of invaginations of the nuclear membrane (Muranyi et al., 2002; Milbradt et al., 2007; Camozzi et al., 2008; Villinger et al., 2015).

The data described above for all these pathologies emphasize that the processing of lamins plays an essential role in the formation of NR structures.

Medication for the treatment of certain diseases may also have an effect on the organization of the NR. A recent study showed that autophagy induction as a result of DNA damage induced by DNA-damaging agents used in chemotherapy is also associated with NR formation. Autophagy led to the removal of genotoxin-induced micronuclei-like structures and protected the cell against genotoxin-induced cell death. The autophagic receptor P62/SQSTM1 was found to be clustered in the genotoxin-induced NR, suggesting a role of NR formation in pro-survival autophagy (He et al., 2021).

**FIGURE 7:**
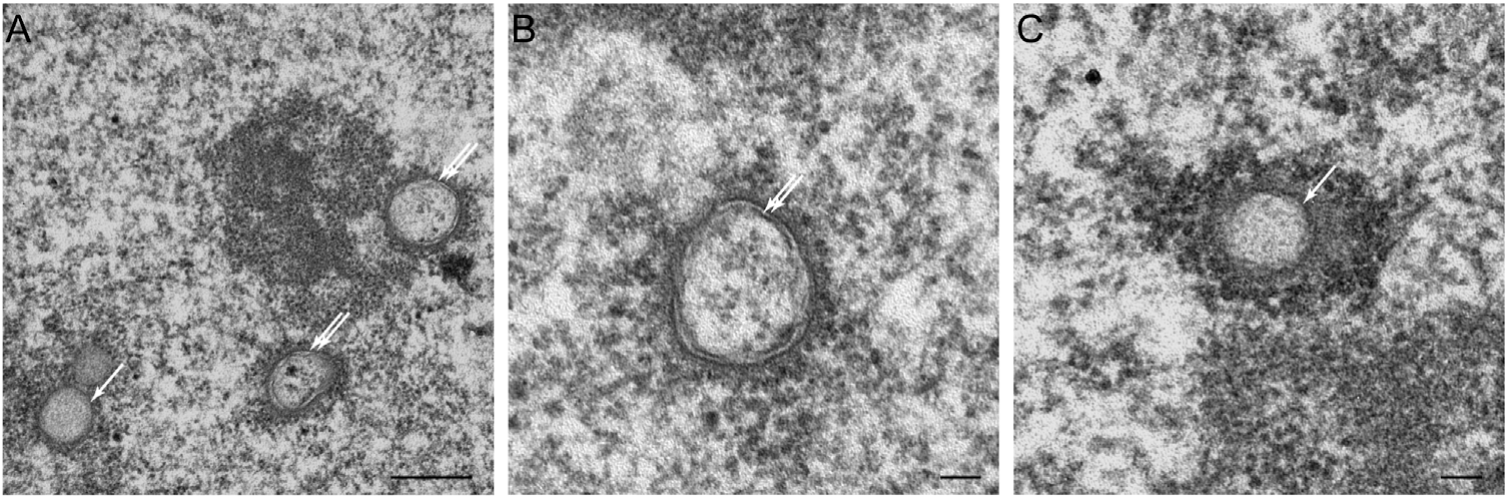
The effects of lovastatin treatment on the nucleus of CHO-cells at the electron microscopy (EM)-level. **(A)** EM recording of two types of tubular invaginations in CHO cells treated with lovastatin. Type I invaginations are marked by a single arrow, type II by a double arrow. Scale bar indicates 0.5 μm. **(B)** Detailed recording of type II NRin CHO cells treated with lovastatin. Scale bar indicates 0.1 μm. **(C)** Detailed recording of a type I NR in CHO cells treated with lovastatin. Scale bar indicates 0.1 μm. Ultrathin sections from fibroblasts of CHO cells were studied by electron microscopy (for sample preperations, see Figure 5) for nuclear membrane invaginations after 18 h of incubation with 40 μM of lovastatin (see also Verstraeten et al., 2006).

Statins, also known as HMG-CoA reductase inhibitors, are a class of lipid-lowering medications that reduce risk for illnesses related to atherosclerosis and mortality in those who are at high risk of cardiovascular disease. When treated with lovastatin, CHO cells exhibited two types of NR invaginations, i.e., type II NR (Figures 7A,B) identical to the invaginations seen in cells which over-express lamin A (Figures 3F,G), and type I NR (Figures 7A,C). The latter are not seen in cells that over-express lamin A. In these cells, the single membrane invaginations have a smaller diameter (208 ± 26 nm) than the double membrane invaginations (326 ± 26 nm). The formation of nuclear invaginations as a results of lovastatin treatment has been reported before in patient fibroblasts (Verstraeten et al., 2006).

In conclusion, the pleiomorphic NR is a highly flexible structure involved in many cell biological processes, both in normal and diseased cells and tissues. Although multiple hypothesis have been formed, the exact formation of the NR remains to be elucidated. It is not unlikely that multiple mechanisms are involved, since many different proteins seem to be involved in NR formation. Specifically, the processing of lamins is of high importance. Similar to the formation, exact mechanisms related to the wide variety of functions ascribed to the NR warrant further investigation.
