# Supplementary material for: The Role of Lamins in the Nucleoplasmic Reticulum, a Pleiomorphic Organelle That Enhances Nucleo-Cytoplasmic Interplay
Source: Front Cell Dev Biol. 2022 Jun 16;10:914286. doi: 10.3389/fcell.2022.914286 (PMC9243388; doi:10.3389/fcell.2022.914286)
Supplement: Supplementary file 1 [file DataSheet1.PDF]

# *Supplementary Material*

## 1.1 Supplementary Figures

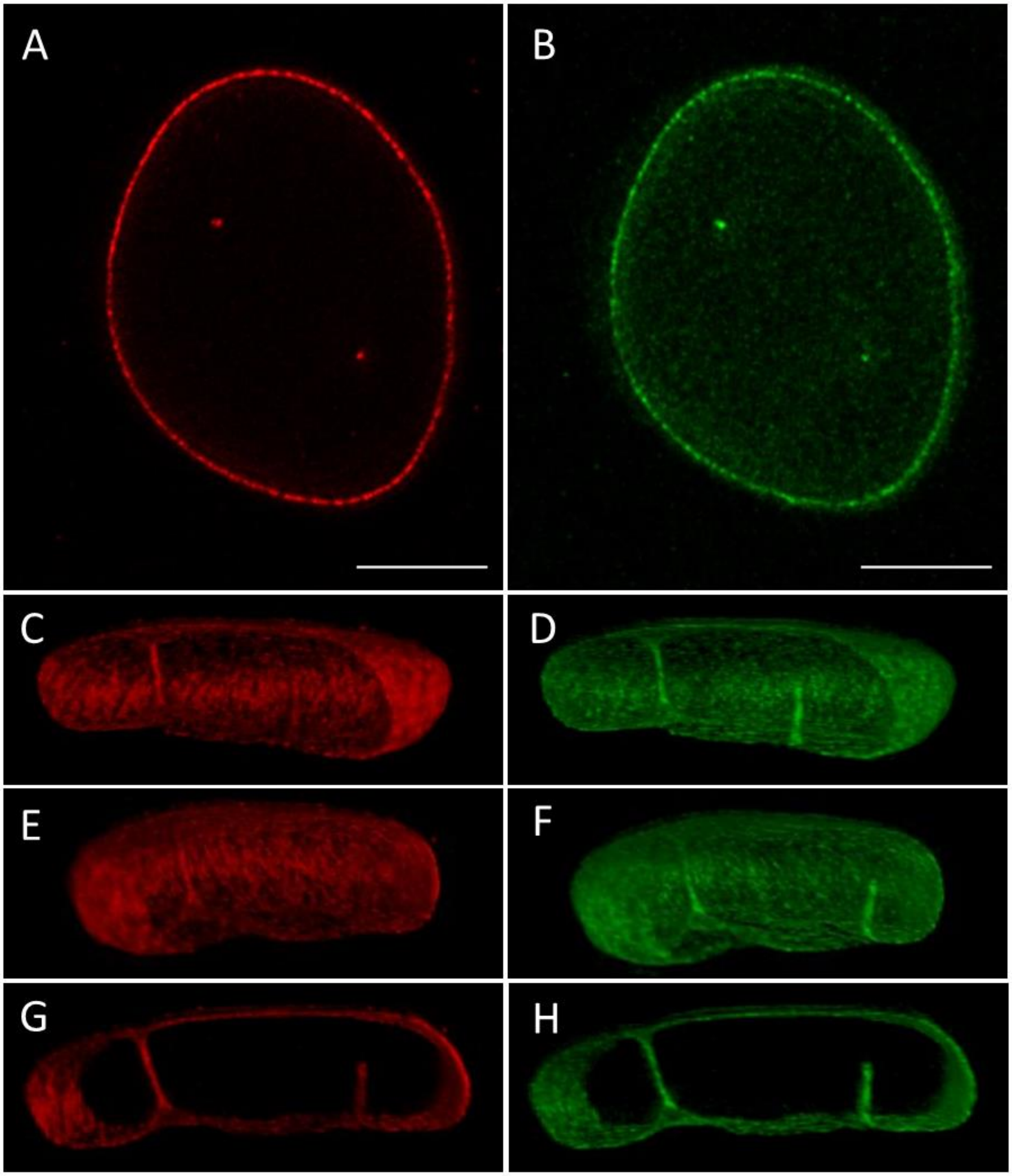


**Supplementary Figure 1:** 3D STED of normal human dermal fibroblast cells, stained for lamin A/C or B1. **(A-B)** 2D image of the middle of the nucleus stained for lamin A/C **(A)** or lamin B1 **(B)**. **(C-H)** Different 3D reconstruction views of the cell in **(A)** and **(B)** respectively using ImageJ 3D viewer (lamin A displayed in red, lamin B in green). Scale bars indicate 5 µm.

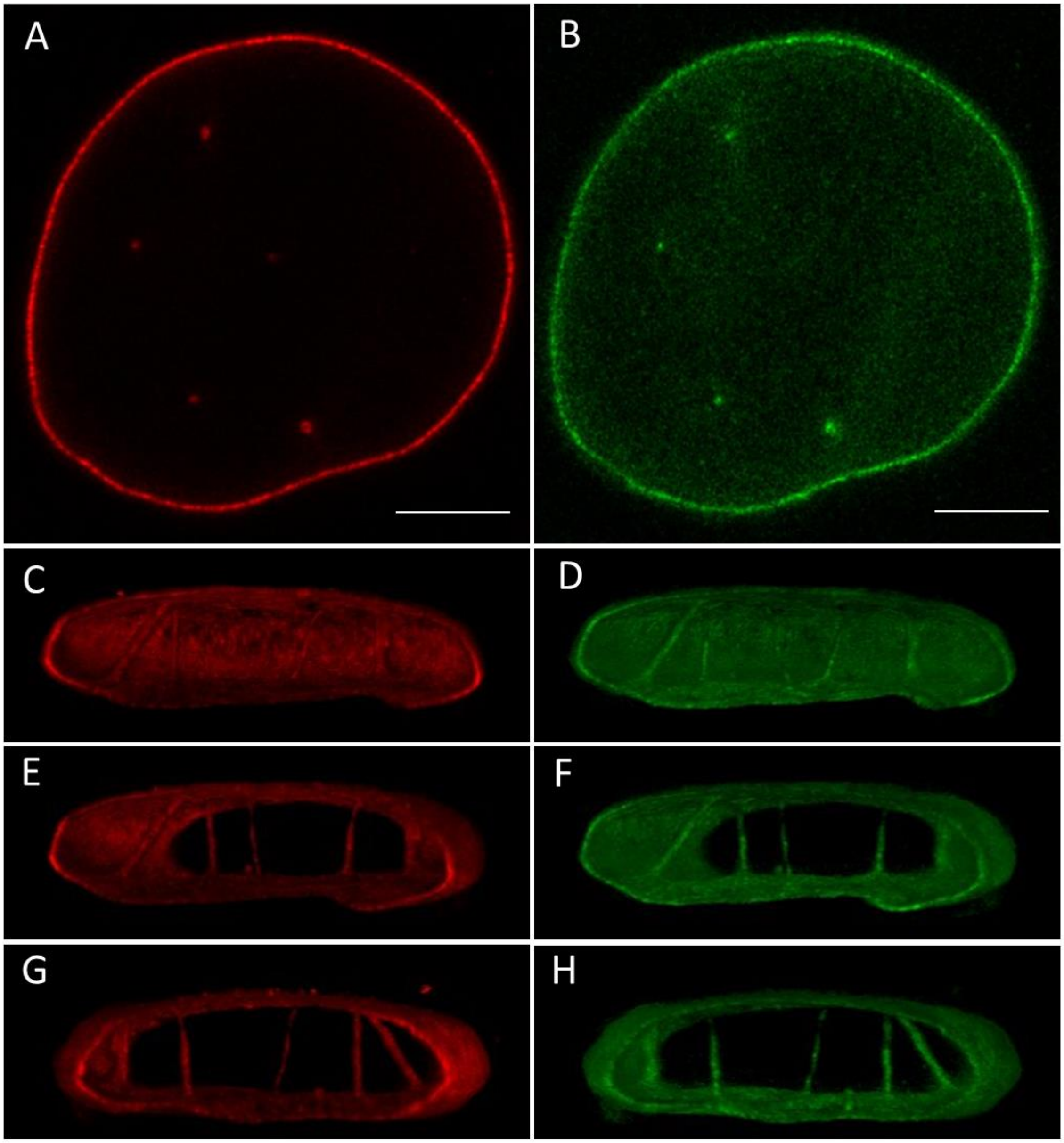


**Supplementary Figure 2:** 3D STED of normal human dermal fibroblast cells, stained for lamin A/C or B1. **(A-B)** 2D image of the middle of the nucleus stained for lamin A/C **(A)** or lamin B1 **(B)**. **(C-H)** Different 3D reconstruction views of the cell in **(A)** and **(B)** respectively using ImageJ 3D viewer (lamin A displayed in red, lamin B in green). Scale bars indicate 5 µm.
